# A new species of the leafhopper genus *Diomma* Motschulsky (Hemiptera, Cidadellidae, Typhlocybinae) from China

**DOI:** 10.3897/zookeys.83.1177

**Published:** 2011-02-25

**Authors:** Yuehua Song, Zizhong Li, Jing Xiong

**Affiliations:** 1 Institute of Entomology, Guizhou University, Guiyang, Guizhou 550025, China; 2 Institute of South China Karst, Guizhou Normal University, Guiyang, Guizhou 550001, China

**Keywords:** Hemiptera, Auchenorrhyncha, new species, morphology, taxonomy, China

## Abstract

In the present paper, a new species is added to the genus Diomma Motschulsky (Hemiptera: Cicadellidae: Typhlocybinae) from Southwest China, Diomma pincersa **sp. n.** At the same time, a key can distinguish all Chinese species of the genus is provided.

## Introduction

The leafhopper genus Diomma was established by Motschulsky in 1863 (Dworakowska 1981; Chiang and Knight 1990). Diomma belongs to the tribe Erythroneurini (Typhlocybinae) with Diomma ochracea Motschulsky, 1863 as its type species. The genus consists of three subgenera: Diomma Motschulsky; Bunyipia Dworakowska and Dilobonota Dworakowska. So far, all species occurring in China belong to the subgenus Diomma, which distributed only in Oriental region. A new species from Guizhou Province, China is described and illustrated. A key to Chinese species of Diomma is given. All specimens examined are deposited to the collection of the Insititute of Entomology, Guizhou University, Guiyang, China (GUGC).

## Taxonomy

### Diomma

Motschulsky

Diomma Motschulsky 1863: 102

#### Type species:

Diomma ochracea Motschulsky, 1863.

#### Description.

Body yellow or brownish yellow, more or less flattened. Head almost equally broad as pronotum. Crown anterior margin produced medially. Pronotum with width greater than length. Vertex and pronotum usually ornamented with dark spots or stripes. Scutellum small, triangular; transverse impression distinct. Forewing apical veins free or 3rd apical cell stalked; 4th apical cell smallest. Hind venation reduced, submarginal vein poorly developed.

Abdominal apodemes long and narrow.

Pygofer large and broad, with numeous long setae at caudal margin and baso-ventral angle respectively. Subgenital plate extending beyond pygofer, with several microsetae on dorsal margin and with few of long macrosetae on outer surface. Pygofer dorsal appendage with distinct basal suture, but not movably articulated or immovably fused to margin, without basal suture. Central part of style very thick; preapical lobe prominant, sensory pits situated at preapical portion. Aedeagal shaft curved ventrally, usually with a obvious big process between preatrium and base of shaft. Gonopore terminal or subapical. Connective V- or Y-shaped; two lateral arms very long; central lobe absent or vestigial.

#### Distribution.

Afrotropical region, Australian region, Oriental region.

#### Key to Chinese species (♂) of the genus Diomma.

**Table:** 

1	Aedeagus preatrium with a large preatrial process	2
–	Aedeagus preatrium without preatrial process (Fig. 18)	Diomma pulchra (Matsumura, 1916)
2	Gonopore apical	3
–	Gonopore subapical	5
3	Aedeagal shaft with paired processes medially, which edges serrated (Fig. 19)	Diomma taiwana (Shiraki, 1912)
–	Aedeagal shaft without paired processes medially	4
4	Apex of aedeagal shaft with a short, rigid process (Fig. 17)	Diomma knighti Dworakowska, 1981
–	Apex of aedeagal shaft without short, rigid process (Fig. 16)	Diomma katoi Dworakowska, 1981
5	Aedeagal shaft with wide and compressed processes in median, which lateral margin serrated (Figs 8, 9)	Diomma pincersa sp. n.
–	Aedeagal shaft without process in median (Fig. 14)	Diomma ilsae (Jacobi, 1941)

### Diomma (Diomma) pincersa

Song, Li & Xiong sp. n.

urn:lsid:zoobank.org:act:32756A97-957D-4B3E-8D11-398522C61F34

Figures 1–10 

#### Description.

**Figures 1–10.**
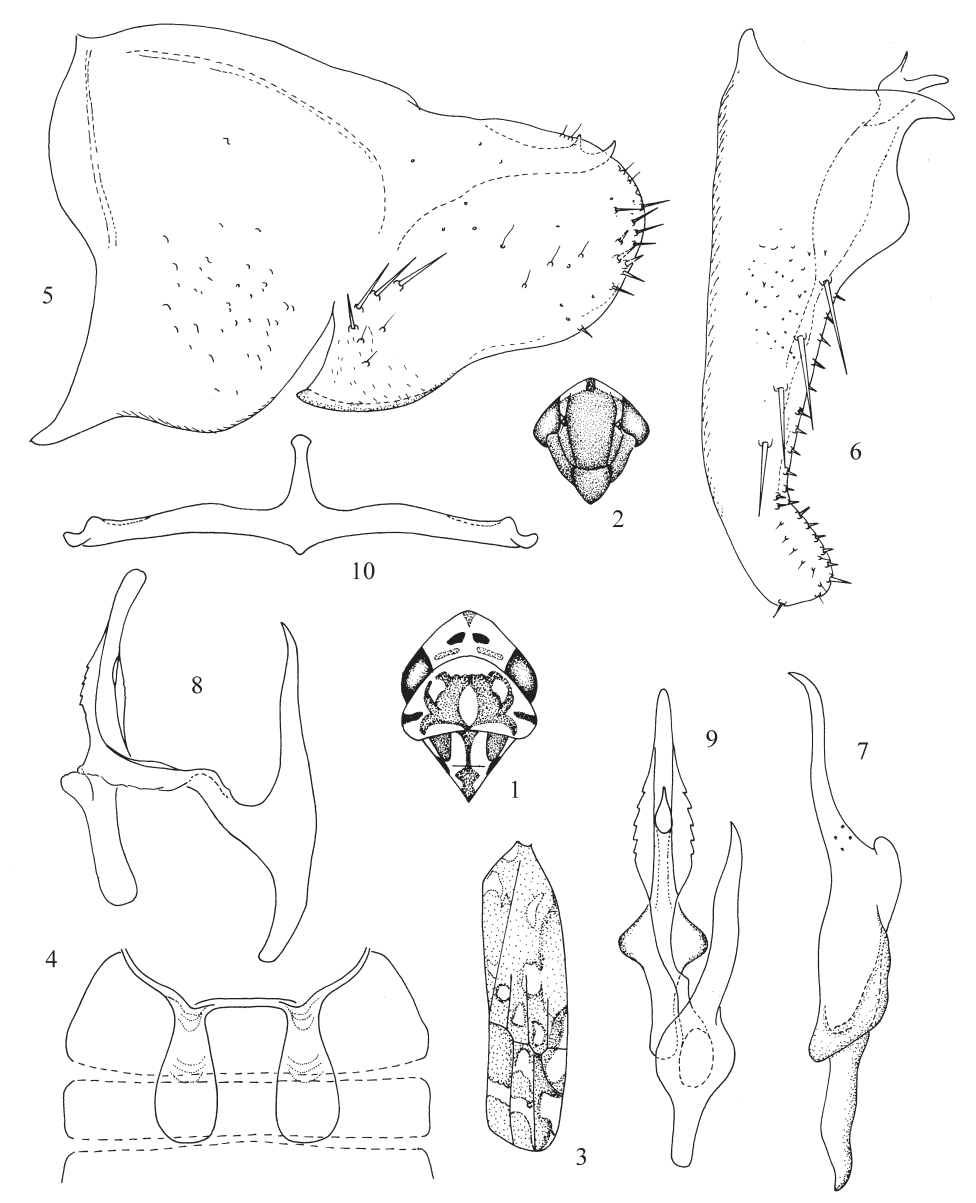
Diomma (Diomma) pincersaSong, Li & Xiong, sp. n. **1** Head and thorax, dorsal view **2** Face **3** Fore wing **4** Abdominal apodemes **5** Pygofer lobe, lateral view **6** Subgenital plate **7** Style **8** Aedeagus, lateral view **9** Aedeagus, ventral view **10** Connective.

Head (Fig. 1) width about equal in length to greast width of pronotum. Vertex (Fig. 1) yellow, with a large median apical spot, orange red and with four irregular spots: one pair near posterior margin of crown light brown; other pair smaller, blackish brwon. Eyes (Figs 1, 2) greyish black. Face (Fig. 2) brownish black, with a orange red spot at its upper part. Anteclypeus quite large and broad, little expanded, nearly pentagonal. Pronotum (Fig. 1) with large irregular nut-brown stripes. Scutellum (Fig. 1) small, triangular; basal triangles blackish brown, a longitudinal dark stripe extended from base to apex. Forewing wax field orange yellow, with several irregular markings as in Fig. 3.

Abdominal apodemes (Fig. 4) extended to posterior margin of 4th sternite.

Pygofer lobe (Fig. 5) broad, with few of long macrosetae on lateral surface. Pygofer dorsal appendage immovably fused to margin, its terminal part bifurcate. Subgenital plate (Fig. 6) long, much protruding beyond pygofer lobe, with four basal macrosetae and numerous short rigid setae along upper margin. Style (Fig. 7) broad in middle, long and slender at apical portion, with four sensory pits not far from preapical lobe. Preapical lobe small, but prominent. Aedeagal shaft (Figs 8, 9) curved ventrally, with serrated lateral margin in median; shaft elongated. Preatrium with a large process, its length about as long as that of dorsal apodeme. Gonopore subapical, ventrad. Connective (Fig. 10) Y-shaped, two lateral arms very long, which more than two times of connective stem length; central lobe small, quite vestigial.

#### Measurement.

Body length males 2.8 mm.

#### Type material.

*Holotype*, male, China: Guizhou Province, Qianxi County, 15~17 October 2007, coll. QIONG-ZHANG SONG. *Paratype*: one male, same date as holotype.

#### Remarks.

The new species can be distinguished from other species of the genus by its unique aedeagus’s structure (Figs 8, 9).

#### Etymology.

The specific name is derived from the Latin word “pincersa” (claw, clamp), which refers to the pygofer dorsal appendage with terminal part branched or bifurcate (Fig. 5).

### Diomma (Diomma) ilsae

(Jacobi, 1941) rec. n.

Figures 11–15

Typhlocyba ilsae Jacobi 1941
Zyginoides ilsae Dworakowska 1972: 860
Diomma ilsae Dworakowska 1981: 364

#### Type material.

six males, six females, China: Yunnan Province, Mengla County, 18 July 2008, coll. Yuehua Song; one female, China: Yunnan Province, Menghai County, 24 July 2008, coll. Yuehua Song.

**Figures 11–15.**
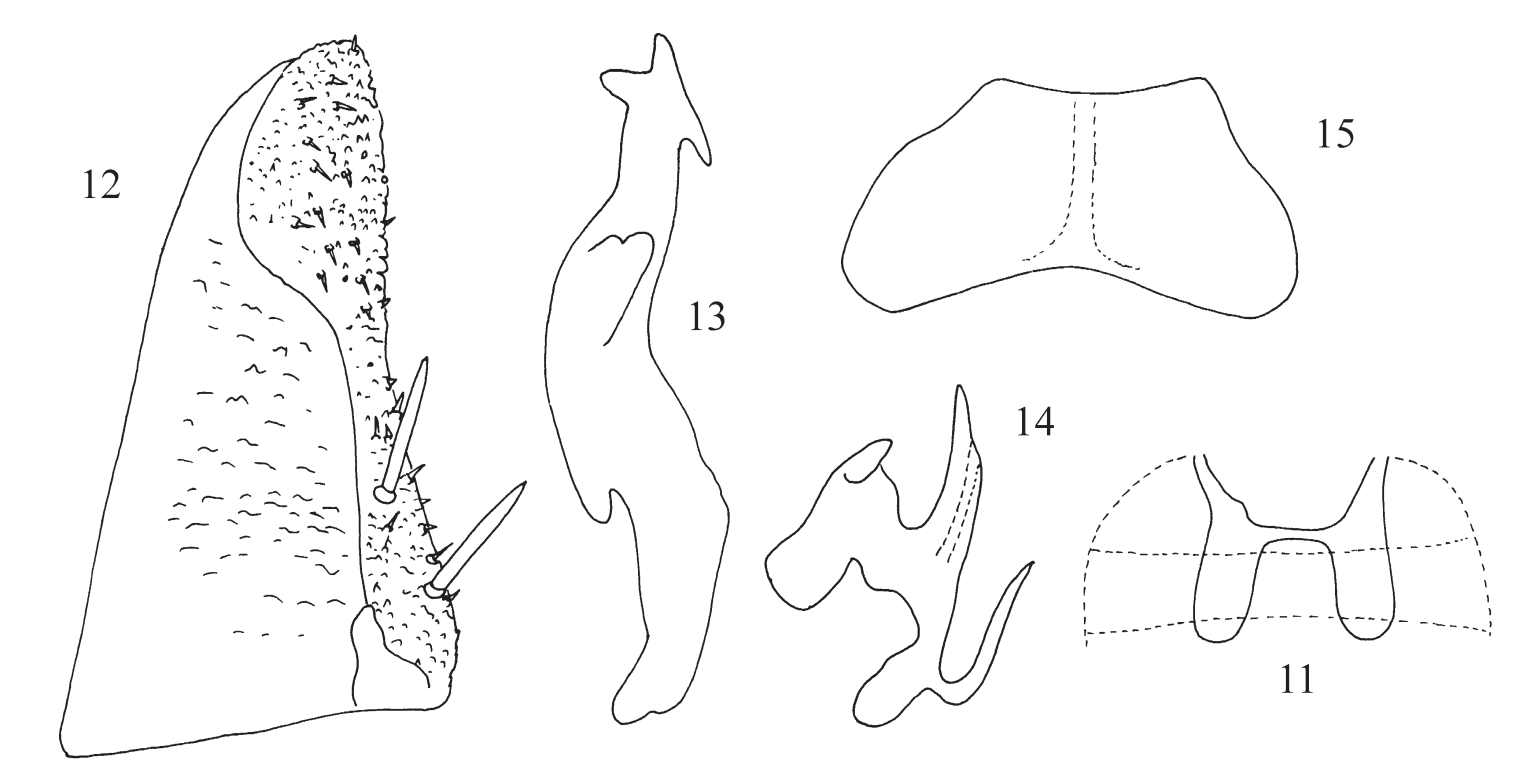
Diomma (Diomma) ilsae Jacobi, 1941, rec. n. (after Dworakowska, 1972) **11** Abdominal apodemes **12** Subgenital plate **13** Style **14** Aedeagus, lateral view **15** Connective.

**Figures 16–19.**
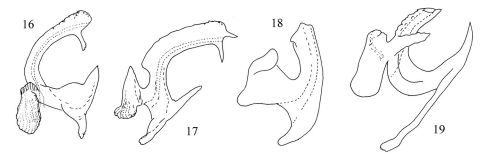
Aedeagus, lateral view of Diomma species. **16** Diomma (Diomma) katoi Dworakowska, 1981 **17** Diomma (Diomma) knighti Dworakowska, 1981 **18**Diomma (Diomma) pulchra (Matsumura, 1916) **19** Diomma (Diomma) taiwana (Shiraki, 1912) (all figures after Dworakowska, 1972; 1981).

#### Distribution.

Sunda; China (Yunnan).

## Species checklist of Diomma from China

Diomma (Diomma) ilsae (Jacobi 1941), rec. n.

Typhlocyba ilsae Jacobi, 1941

Zyginoides ilsae Dworakowska 1972

Diomma ilsae Dworakowska, 1981

Distribution: China (Yunnan: Mengla, Menghai); Sunda

Diomma (Diomma) katoi Dworakowska 1981

Distribution: China (Taiwan: Taipei; Guizhou: Rongjiang)

Diomma (Diomma) knighti Dworakowska 1981

Distribution: China (Taiwan: Chiayi; Guizhou: Bijie)

Diomma (Diomma) pincersa Song, Li & Xiong, sp. n.

Distribution: China (Guizhou: Qianxi)

Diomma (Diomma) pulchra (Matsumura 1916)

Motschulskia pulchra Matsumura 1916

Platytettix pulchrus Matsumura 1932

Zyginoides (Platytetticis) pulchra Dworakowska 1972

Diomma pulchra Dworakowska 1981

Distribution: China (Taiwan: Taichung; Guizhou: Guiyang, Yanhe); Japan

Diomma (Diomma) taiwana (Shiraki 1912)

Eupteryx taiwanus Shiraki 1912

Zygina bokotonis Matsumura 1932

Pakeasta notata Ahmed 1971

Diomma taiwana Dworakowska 1981

Distribution: China (Taiwan: Taipei, Chiayi; Guizhou: Luodian, Xingyi; Yunnan: Pu’er; Hainan: Haikou); Japan; India

## Supplementary Material

XML Treatment for Diomma

XML Treatment for Diomma (Diomma) pincersa

XML Treatment for Diomma (Diomma) ilsae
